# Identification of Prognostic and Metastatic Alternative Splicing Signatures in Kidney Renal Clear Cell Carcinoma

**DOI:** 10.3389/fbioe.2019.00270

**Published:** 2019-10-15

**Authors:** Tong Meng, Runzhi Huang, Zhiwei Zeng, Zongqiang Huang, Huabin Yin, ChenChen Jiao, Penghui Yan, Peng Hu, Xiaolong Zhu, Zhenyu Li, Dianwen Song, Jie Zhang, Liming Cheng

**Affiliations:** ^1^Division of Spine, Department of Orthopedics, Tongji Hospital affiliated to Tongji University School of Medicine, Shanghai, China; ^2^Key Laboratory of Spine and Spinal cord Injury Repair and Regeneration, Tongji University, Ministry of Education, Shanghai, China; ^3^Department of Orthopedics, School of Medicine, Shanghai General Hospital, Shanghai Jiaotong University, Shanghai, China; ^4^Department of Orthopedics, The First Affiliated Hospital of Zhengzhou University, Zhengzhou, China; ^5^Department of Pathology, Shanghai Tenth People's Hospital, Tongji University School of Medicine, Shanghai, China; ^6^Department of Prevention, Tongji University School of Medicine, Tongji University, Shanghai, China

**Keywords:** alternative splicing, kidney renal clear cell carcinoma, prognosis, tumor metastasis, RHOT2, TCIRG1

## Abstract

**Background:** Kidney renal clear cell carcinoma (KIRC) is the malignancy originated from the renal epithelium, with a high rate of distant metastasis. Aberrant alternative splicing (AS) of pre-mRNA are widely reported to be involved in the tumorigenesis and metastasis of multiple cancers. The aim of this study is to explore the mechanism of alternative splicing events (ASEs) underlying tumorigenesis and metastasis of KIRC.

**Methods:** RNA-seq of 537 KIRC samples downloaded from the TCGA database and ASEs data from the TCGASpliceSeq database were used to identify ASEs in patients with KIRC. The univariate and Lasso regression analysis were used to screen the most significant overall survival-related ASEs (OS-SEs). Based on those, the OS-SEs model was proposed. The interaction network of OS-SEs and splicing factors (SFs) with absolute value of correlation coefficient value >0.750 was constructed by Pearson correlation analysis. The OS-SEs significantly related to distant metastasis and clinical stage were identified by non-parametric test, and those were also integrated into co-expression analysis with prognosis-related Kyoto Encyclopedia of Genes and Genomes (KEGG) pathways identified by Gene Set Variation Analysis (GSVA). ASEs with significance were selected for multiple online database validation.

**Results:** A total of prognostic 6,081 overall survival-related ASEs (OS-SEs) were identified by univariate Cox regression analysis and a prediction model was constructed based on 5 OS-SEs screened by Lasso regression with the Area Under Curve of 0.788. Its risk score was also illustrated to be an independent predictor, which the good reliability of the model. Among 390 identified candidate SFs, DExD-Box Helicase 39B (DDX39B) was significantly correlated with OS and metastasis. After external database validation, Retained Intron of Ras Homolog Family Member T2 (RHOT2) and T-Cell Immune Regulator 1 (TCIRG1) were identified. In the co-expression analysis, overlapped co-expression signal pathways for RHOT2 and TCIRG1 were sphingolipid metabolism and N-glycan biosynthesis.

**Conclusions:** Based on the results of comprehensive bioinformatic analysis, we proposed that aberrant DDX39B regulated RHOT2-32938-RI and TCIRG1-17288-RI might be associated with the tumorigenesis, metastasis, and poor prognosis of KIRC via sphingolipid metabolism or N-glycan biosynthesis pathway.

## Introduction

Kidney renal clear cell carcinoma (KIRC) is a malignant cancer originated from renal epitheliums, accounting for about 75% of kidney tumors (Hsieh et al., 2017). Therapeutically, although radical nephrectomy is performed for localized renal masses, distant metastasis may be observed in a large proportion of patients at diagnosis, especially metastasis in lung, bone and brain (Gupta et al., 2008). With regard to these advanced KIRCs, the treatment option was limited with only sunitinib widely approved (Porta et al., 2019). Even systematic therapy were applied, including immunotherapeutic agents, antiangiogenic agents and mTOR inhibitors, the prognosis was still poor (Jonasch et al., 2014; Jonasch, 2018). In order to prolong the overall survival of patients with KIRC, there is a pressing need to explore its pathogenic mechanism and identify the potential therapeutic targets related to tumorigenesis, metastasis and prognosis.

Nowadays, most studies of KIRC focused on alteration of transcriptome level and the posttranscriptional process was largely underestimated. Alternative splicing (AS), plays an important role in the maturation of mRNAs from its precursors, leading to diverse mRNA isoforms spliced and protein variants translated (Montes et al., 2019). In this process, splicing factors (SFs) work as regulatory catalyst of alternative splicing events (ASEs) and both build up an intricate regulatory network (Frankiw et al., 2019; Wu et al., 2019). Functionally, AS has been reported to take part in cell differentiation, lineage determination and tissue-specificity acquisition (Wang et al., 2008). The aberrant AS of some genes and somatic mutations of SFs, which make network dysregulated, have been shown to modulate malignant transformation of cells and epithelial-mesenchymal transition (EMT) (Sveen et al., 2016; Kouyama et al., 2019; Wu et al., 2019; Xing et al., 2019). Thus, identifying the dysregulated network may shed light upon the molecular biomarkers for prognosis, metastasis, and potential therapeutic targets (Lee and Abdel-Wahab, 2016; Zhou et al., 2016; Wang et al., 2019).

Nowadays, although a systematic analysis of ASEs was unveiled in KIRC, the regulatory network of ASEs and SFs was not explored (Song et al., 2019). Additionally, metastasis-related ASEs, and potential therapeutic targets were also underestimated. In this study, we performed a comprehensive analysis of AS profiling to identify the overall survival-related ASEs (OS-SEs) in patients with KIRC and construct a prognostic model. Additionally, metastasis-related ASEs along with corresponding SFs and pathways were also identified by Pearson correlation analysis to illuminate the underlying mechanism of metastasis in KIRC. The prediction model might assist oncologists in clinical decision-making. Moreover, we also identified a new candidate molecular mechanism and two potential therapeutic targets for KIRC metastasis treatment, especially to the bone metastasis.

## Materials and Methods

### Data Collection

Clinical information, RNA sequencing profiles, and SFs of KIRC samples were collected from the Cancer Genome Atlas (TCGA) database (https://portal.gdc.cancer.gov). Meanwhile, ASEs data were retrieved from the TCGASpliceSeq database (https://bioinformatics.mdanderson.org/TCGASpliceSeq/) (Ryan et al., 2016) including seven types (alternate acceptor site, AA; exon skip, ES; alternate terminator, AT; mutually exclusive exons, ME; retained intron, RI; alternate donor site, AD; alternate promoter, AP) (Chen et al., 2019). Samples with more than 25 percent of missing percent splicing (PSI) values were excluded. The ASE was presented with gene name, ID number from the TCGASpliceSeq database (AS ID) and splicing pattern.

### The Identification of OS-SEs

The K-Nearest Neighbor algorithm was performed to impute ASEs with missing expression data. ASEs, whose means and standard deviations of PSI < 0.05 and 0.01, were excluded, neither were samples without follow-up records. Then, the combined ASEs along with clinical data were put into the univariate Cox regression analysis to evaluate the prognostic value of each filtered ASE. The UpSet plot was developed to illustrate OS-SEs and volcano plot was used to display the prognosis-related and -unrelated ASEs integrally. The bubble plots were generated to present the top 20 OS-SEs for seven types of ASEs, in which the color and size of bubbles symbolize the value of ASEs for overall survival.

### The Construction of the Prognostic Model Based on the OS-SEs

The Lasso regression was firstly performed to screen the top 20 significant prognostic OS-SEs and then the significant prognostic OS-SEs were evaluated by the multivariate Cox regression model with β value, which represented the regression coefficient of each integrated OS-SE in the model. Risk score was thus acquired by the following formula:

$$\begin{array}{l} \overset{n}{\underset{i=1}{\sum }}\beta \text{i}\times \text{PSI} \end{array}$$

According to the median risk score, samples were divided into two risk groups medially. The area under receiver operating characteristic ROC curve was used to evaluate the accuracy of the model. In addition, Kaplan-Meier survival analysis was also conducted to compare the difference between high- and low-risk group. Samples were reordered according to risk score and then the risk curve, scatterplot and expression heatmap were generated.

The univariate and multivariate Cox regression analysis, modified by baseline information, were applied to evaluate the prognostic role of risk score, along with age, gender, grade, clinical stage, and TNM stage.

### The Construction of the Interaction and Correlation Network

In the SpliceAid2 database, 390 splicing factors were retrieved (Piva et al., 2009). Pearson correlation analysis was performed to explore the interaction and correlation between SFs and OS-SEs. The regulation network of SFs and OS-SEs was plotted by Cytoscape (3.7.1) (Shannon et al., 2003), in which the regulation pairs with P > 0.001 and the absolute value of correlation coefficient < 0.750 were excluded. In the network, we defined SF and OS-SEs as arrows and ellipses, high and low risk of OS-SEs as red and purple, positive and negative regulations as red and green lines, respectively.

### The Identification of Metastasis- and/or Stage-Related OS-SEs

To identify the OS-SEs related to metastasis and/or TNM stage, we performed Kruskal-Wallis test and Mann-Whitney-Wilcoxon test, which were displayed by beeswarm plots. Besides, the regulation network of these metastasis-, and/or stage- related OS-SEs were also explored.

### The Co-expression Analysis Between ASEs and Signaling Pathways

The univariate Cox analysis was performed to screen the prognosis-related signaling pathways identified by Gene Set Variation Analysis (GSVA) (Hänzelmann et al., 2013). Then, metastasis and stage-related OS-SEs and prognosis-related KEGG pathways were put into the co-expression analysis to identify the possible downstream mechanism of OS-SEs.

### Online Database Validation

In order to ensure the roles of selected metastasis and stage-related OS-SEs, multiple databases including the UALCAN (Chandrashekar et al., 2017), UCSC Treehouse Childhood Cancer Initiative, Kaplan Meier plotter (Nagy et al., 2018), LinkedOmics (Vasaikar et al., 2018), SurvExpress (Aguirre-Gamboa et al., 2013) and Firebrowse (Deng et al., 2017) were used to detect their gene and protein expression levels in KIRC and normal kidney tissues.

### Immunohistochemistry (IHC)

The IHC slides and information were obtained from the Human Protein Atlas. Immunostaining on each slide was assessed by experienced pathologists to examine the percentage of RHOT2 and TCIRG1 positive tumor cells and presented as histochemistry score (H-score). H-score = Σpi(i+1) where i is the intensity score and pi is the percent of the cells with that intensity.

### Statistical Analysis

All statistical analysis was applied by R version 3.5.1 (Institute for Statistics and Mathematics, Vienna, Austria; https://www.r-project.org) (Package: impute, UpSetR, ggplot2, rms, glmnet, preprocessCore, forestplot, survminer, survivalROC, beeswarm). For descriptive statistics, mean ± standard deviation was used for the continuous variables in normal distribution while the median (range) was used for continuous variables in abnormal distribution. Categorical variables were described by counts and percentages. Two-tailed *P* < 0.05 was regarded statistically significant.

## Results

### Overview of ASEs and OS-SEs in KIRC

**Figure 1:**
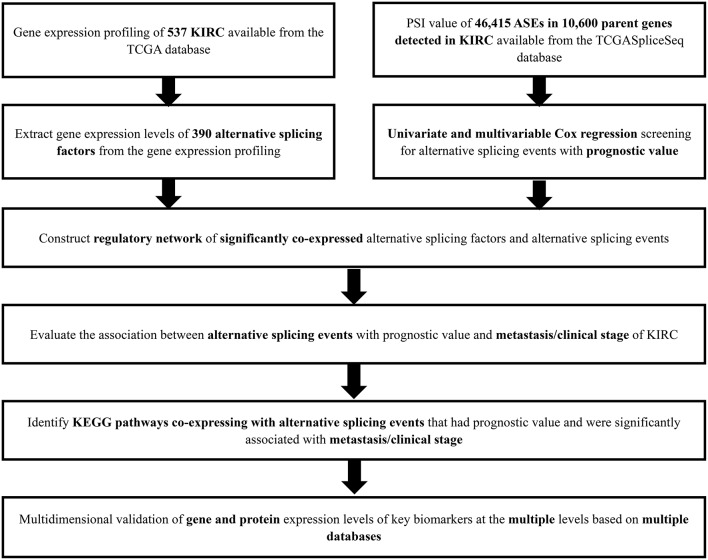
The flowchart of this study.

**Figure 2:**
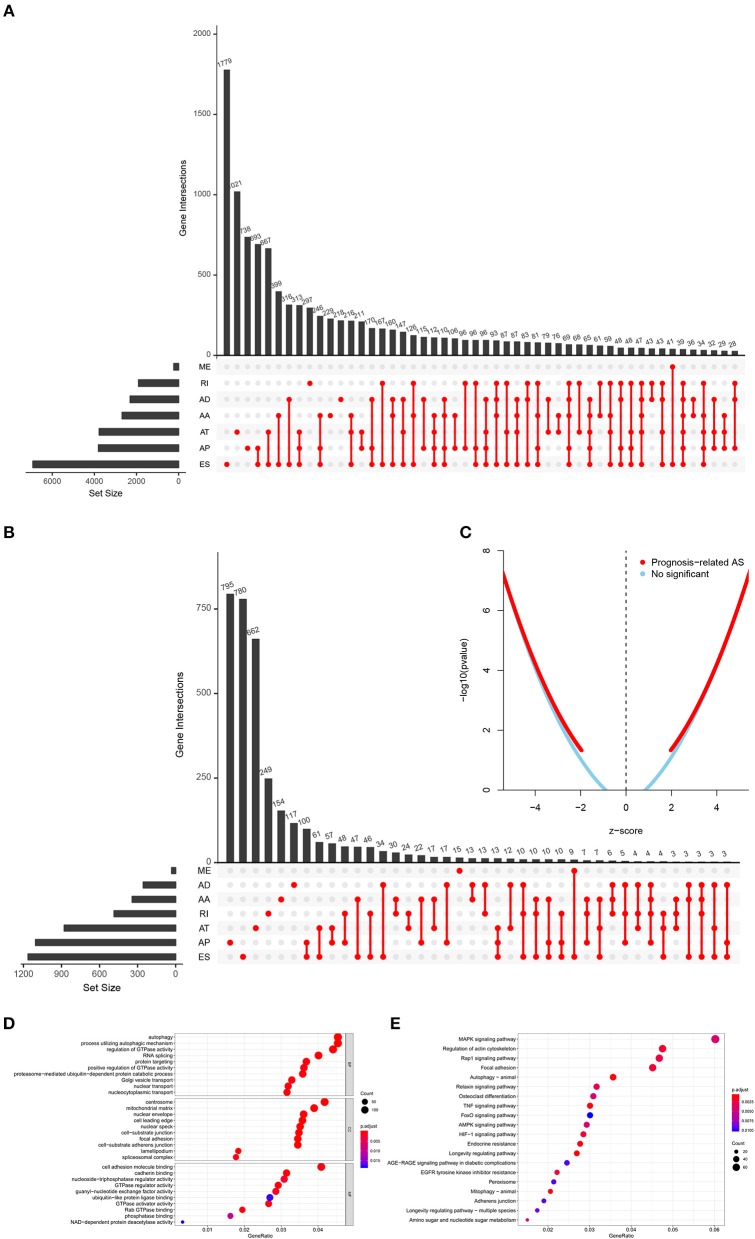
The identification of OS-SEs in KIRC patients. The Upset plots of ASEs and OS-SEs: **(A)** The number of ASEs in different types of splicing patterns; **(B)** The number of OS-SEs in different types of splicing patterns. **(C)** The volcano plot of the prognosis-related and no significant ASEs, respectively; The GO analysis **(D)** and the KEGG pathways enrichment analysis **(E)** of the parent genes of OS-ASEs. ASEs, Alternative splicing events; OS-SEs, overall survival-related ASEs; KIRC, kidney renal clear cell carcinoma; AA, alternate acceptor; AD, alternate donor; AP, alternate promoter; AT, alternate terminator; ES, exon skip; ME, mutually exclusive exons; RI, retained intron; GO, Gene Ontology; KEGG, Kyoto Encyclopedia of Genes and Genomes.

The analysis process was presented in the flow chart (Figure 1). The sequencing data of 537 cases KIRC were downloaded from the TCGA database, with the median overall survival of 1,091 (range, 0–3,668) days. Throughout the follow-up period, 165 patients died and 496 experienced tumor metastases. A total of 46,415 ASEs in 10,600 parent genes were detected in patients with KIRC, including 3,821 AAs (2,683 genes), 3,270 ADs (2,300 genes), 9,509 APs (3,805 genes), 8,632 ATs (3,770 genes), 18,117 ESs (6,915 genes), 235 MEs (227 genes), and 2,831 RIs (1,902 genes). Thus, one gene could undergo more than 4 splicing patterns (Figure 2A). Among the seven types of ASEs, ES was the most prevalent one, followed by AT. A total of 6,081 OS-ASEs from 3,444 parent genes were identified and the UpSet plot revealed that AP was the most common splicing patterns associated with KIRC prognosis (Figure 2B). The volcano plot suggested that most of ASEs were OS-SEs in KIRC (Figure 2C). The top 20 OS-ASEs in seven types of splicing patterns were illustrated in bubble plots (Supplementary Figures 1A–G).

### Functional Enrichment Analysis of Prognostic AS Events

In order to illuminate the potential mechanism underlying the OS-ASEs, 2,077 parent genes of the 6,081 OS-ASEs in KIRC were sent for Gene Ontology (GO) and Kyoto Encyclopedia of Genes and Genomes (KEGG) enrichment analysis (Figures 2D,E). The biological process of GO analysis revealed the enrichment of some well-known pathways, in relation to “autophagy”, “process utilizing autophagic mechanism” and “regulation of GTPase activity” (Figure 2D). Besides, the RNA splicing was also enriched significantly, which meant the active aberrant splicing patterns of KIRC. Additionally, “centrosome” “mitochondrial matrix” and “cell adhesion molecule binding” were also significantly enriched as cellular component or molecular function. The KEGG enrichment analysis suggested some key pathways were associated with the OS of patients with KIRC, such as “MAPK signaling pathway”, “Regulation of actin cytoskeleton”, “Rap1 signaling pathway” and “Focal adhesion” (Figure 2E).

### Establishment of the Prediction Model

**Figure 3:**
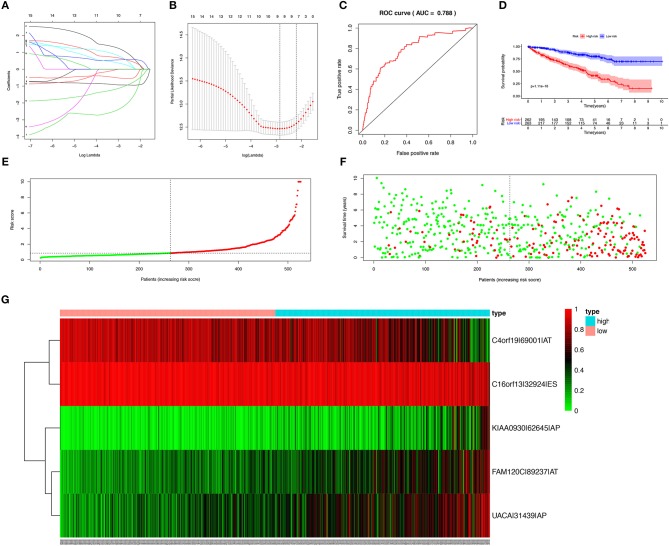
Establishment and assessment of the predict model. **(A)** The coefficients in the Lasso regression for OS-SEs screening; **(B)** Cross-validation for tuning parameter selection in the proportional hazards model; **(C)** The ROC curve for assessing the reliability of the predict model; **(D)** The Kaplan-Meier curve of the predict model; **(E)** The risk curve of each sample reordered by risk score; **(F)** The scatter plot of the samples. The green and red dots representing survival and death, respectively; **(G)** The heatmap of expression level of 5 OS-SEs filtered by Lasso regression. OS-SEs, overall survival-related ASEs; ROC, receiver operating characteristic.

In order to avoid over-fitting of the predict model, the Lasso regression was performed to screen the top 20 OS-SEs. The result showed that C4orf19-69001-AT, C16orf13-32924-ES, KIAA0930-62645-AP, FAM120C-89237-AT, UACA-31439-AP were included in the multivariate Cox regression analysis (Figures 3A,B), with Area Under Curve (AUC) of 0.788 in ROC curve (Figure 3C). Accordingly, risk score of each sample was calculated, with a median value of 0.853. Then, Kaplan-Meier curve revealed that prediction model of risk score had a good effectiveness (*P* < 0.001) (Figure 3D).

Risk curve and scatterplot were generated to show the risk score and vital status of each patient with KIRC. Patient in high-risk group had a higher mortality than patient in low-risk group (Figures 3E,F). The heatmap showed the expressions of OS-SEs screened by Lasso regression, indicating that C40rf19-69001-AT and C16orf13-32924-ES were lower and KIAA0930-62645-AP, FAM120C-89237-AT, UACA-31439-AP were higher in high-risk group (Figure 3G).

### The Risk Score Predicted Prognosis

**Figure 4:**
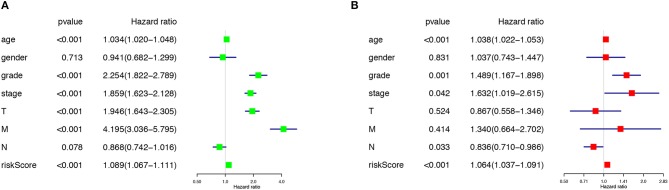
The Cox regression analysis for evaluating the independent prognostic value of the risk score. The univariate **(A)** and multivariate **(B)** Cox regression analysis of risk score, age, gender, grade, and TNM stage.

The risk score along with age, gender, grade and TNM stage were evaluated in the univariate and multivariate Cox regression analysis. The risk score was confirmed as an independent predictor in both univariate (HR = 1.089, 95%CI (1.067–1.111), *P* < 0.001), and multivariate Cox regression analysis (HR = 1.064, 95%CI (1.037–1.091), *P* < 0.001), Figures 4A,B).

The potential splicing regulatory network of SFs and OS-SEs, and their metastasis or clinical stage correlation.

**Figure 5:**
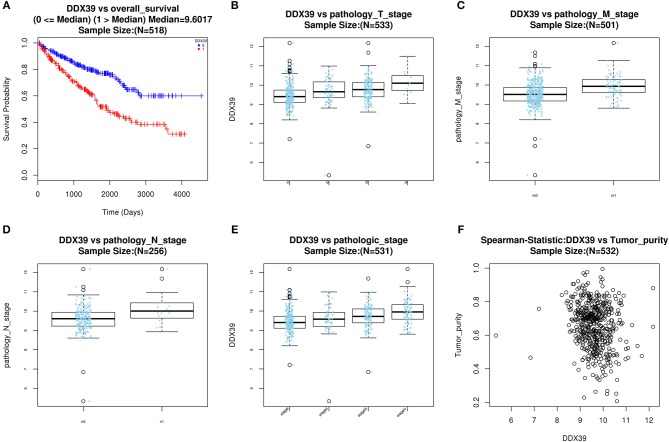
Evaluate prognostic value and clinical correlation of splicing factor DDX39B (Gene symbol: DDX39). **(A)** The Kaplan-Meier curve of DDX39; The expressions of DDX39 according to T **(B)**, N **(C)**, M **(D)** staging system, and clinical stage **(E)**; **(F)** The spearman correlation analysis of DDX39 and tumor purity. DDX39B, DExD-Box Helicase 39B.

**Figure 6:**
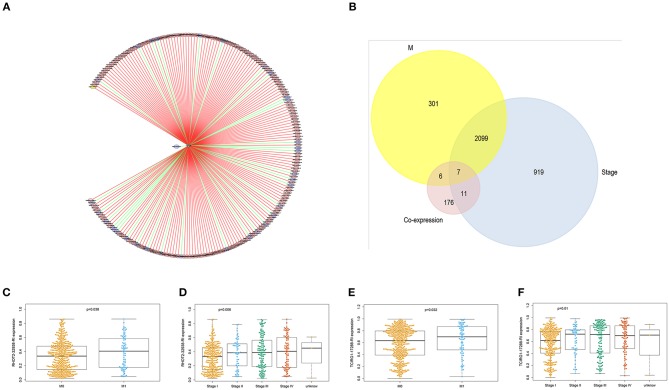
The identification of metastasis- and/or stage-related OS-SEs. **(A)** The network of OS-SEs and prognosis-related SFs; **(B)** The Venn plot to identify the overlapped OS-SEs related to clinical status and metastasis; The beeswarm plots of RHOT2-32938-RI **(C)** and TCIRG1-17288-RI **(D)** according to metastasis or not; The beeswarm plots of RHOT2-32938-RI **(E)**, and TCIRG1-17288-RI **(F)** according to clinical status. OS-SEs, overall survival-related ASEs; SFs, splicing factors; RHOT2, Retained Intron of Ras Homolog Family Member T2; TCIRG1, T-Cell Immune Regulator 1.

With access to RNA-seq data and corresponding clinical information of patients with KIRC, we identified 390 candidate SFs whose expression levels were significantly associated with OS of KIRC patients. Among them, DExD-Box Helicase 39B (DDX39B) was the only SF, who was correlated with prognosis (Figure 5A), TNM staging system (Figures 5B–D), clinical stage (Figure 5E), and tumor purity (Figure 5F). Then, a network was established to demonstrate the interaction and correlation between SFs and OS-SEs. DDX39B was correlated with 34 favorable OS-SEs (purple ellipses) negatively (green lines) and 166 adverse OS-SEs (red ellipses) positively (red lines) (Figure 6A). Among them, 7 OS-SEs (CALCOCO1-22108-RI, CIRBP-46432-RI, P4HTM-64788-ES, RHOT2-32938-RI, TBC1D17-51116-ES, TCIRG1-17288-RI, THOP1-46623-AP) were significantly related to both metastasis and stage in the Venn plot (Figures 6B–F, Supplementary Figures 2A–J).

### External Validation

**Figure 7:**
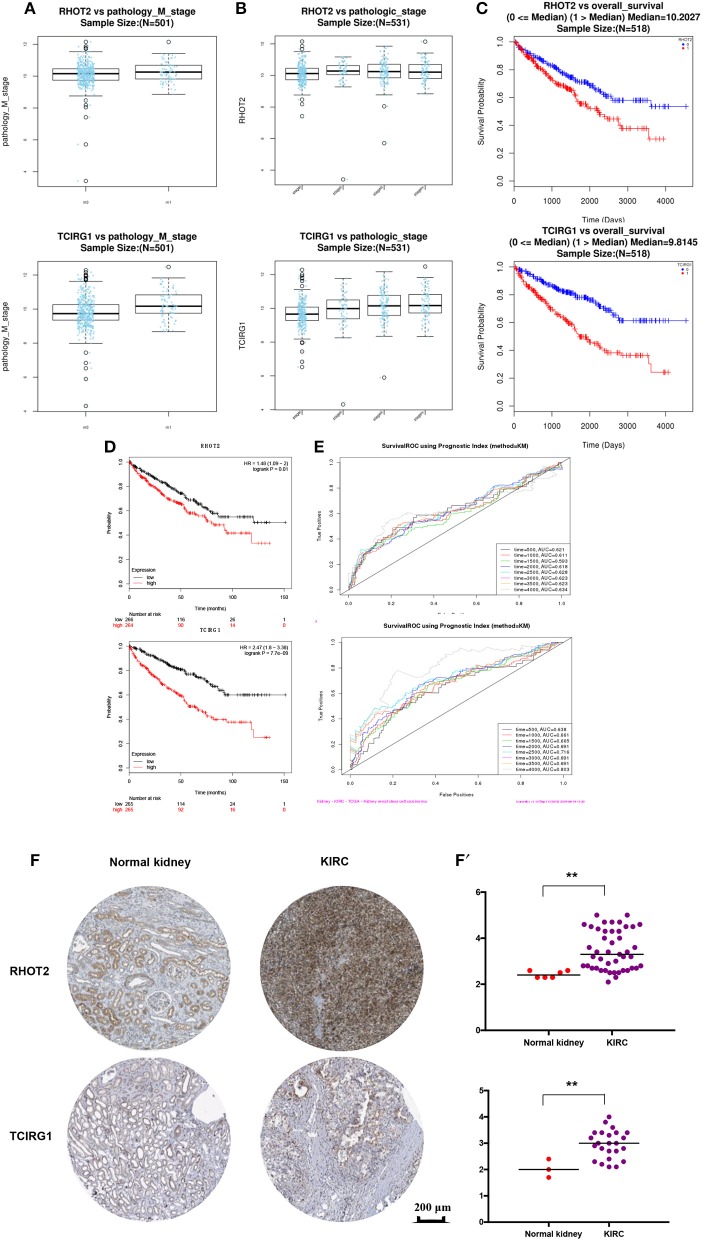
The external validation of RHOT2 and TCIRG1: **(A)** The expressions of RHOT2 and TCIRG1 between normal kidney and KIRC in UALCAN; **(B)** The expressions of RHOT2 and TCIRG1 according to clinical stage in UALCAN; The Kaplan-Meier curve of RHOT2 and TCIRG1 in LinkedOmics **(C)** and Kaplan Meier plotter **(D)**; **(E)** The survicalROC of RHOT2 and TCIRG1; The IHC **(F)** and H-score **(F′)** of RHOT2 and TCIRG1 between normal kidney and KIRC in the Human Protein Atlas. RHOT2, Retained Intron of Ras Homolog Family Member T2; TCIRG1, T-Cell Immune Regulator 1; KIRC, kidney renal clear cell carcinoma; IHC, Immunohistochemistry; H-score, histochemistry score.

The parent genes of the 7 OS-SEs were validated in external databases. RHOT2 and TCIRG1 were confirmed in all the external databases (Table 1). In the database of UALCAN and LinkedOmics, RHOT2, and TCIRG1 were up-regulated in tumor than normal tissue (Figure 7A, Supplementary Figure 3A) and associated with tumor stage (Figure 7B, Supplementary Figure 3B) and OS (Figure 7C, Supplementary Figure 3C) significantly. In the Kaplan Meier plotter and SurvExpress, RHOT2, and TCIRG1 were also associated with OS significantly (Figure 7D, Supplementary Figure 3D). The survicalROC was also described in Figure 7E. Validation in the Human Protein Atlas revealed that the protein levels of RHOT2 and TCIRG1 in KIRC were significantly higher than those in normal kidney tissue (Figures 7F,F′, Table 2).

**Table 1:** The external validation of CALCOCO1, CIRBP, P4HTM, RHOT2, TBC1D17, TCIRG1 and THOP1.

**Database**		**CALCOCO1 (anti-oncogene)**	**CIRBP (anti-oncogene)**	**P4HTM (anti-oncogene)**	**RHOT2 (oncogene)**	**TBC1D17 (oncogene)**	**TCIRG1 (oncogene)**	**THOP1 (oncogene)**
UALCAN		Expression: *p* < 0.001 Stage: *p* < 0.001 K–M analysis: *P* = 0.010	Expression: *p* = 0.023 Stage: *p* < 0.001 K–M analysis: *p* = 0.001	Expression: *p* < 0.001 Stage: *p* < 0.001 K–M analysis: *p* = 0.240	Expression: *p* < 0.001 Stage: *p* < 0.001 K–M analysis: *p* < 0.001	Expression: *p* = 0.010 Stage: *p* < 0.001 K–M analysis: *p* = 0.250	Expression: *p* < 0.001 Stage: *p* < 0.001 K–M analysis: *p* < 0.001	Expression: *p* < 0.001 Stage: *p* < 0.001 K-M analysis: *p* = 0.410
The human protein atlas		Tumor median Normal high K-M analysis *P* < 0.001	Tumor low Normal high K-M analysis *P* < 0.001	Tumor not detected Normal median K-M analysis *P* < 0.001	Tumor high Normal median K-M analysis *P* < 0.001	Tumor median Normal median K-M analysis *P* < 0.001	Tumor high Normal median K-M analysis *P* < 0.001	Tumor high Normal high K-M analysis *P* < 0.001
Kaplan Meier plotter	Best cutoff	*P* < 0.001	*P* < 0.001	*P* = 0.004	*P* < 0.001	*P* < 0.001	*P* < 0.001	*P* = 0.008
	Median value	*P* = 0.334	*P* = 0.121	*P* = 0.168	*P* = 0.011	*P* = 0.216	*P* < 0.001	*P* = 0.193
LinkedOmics		K–M analysis: *P* = 0.364 M: *P* = 0.128 Stage: *P* = 0.047 *R* = 0.541	K–M analysis: *P* = 0.044 M: *P* < 0.001 Stage: *P* < 0.001 *R* = 0.545	K–M analysis: *P* = 0.371 M: *P* = 0.630 Stage: *P* = 0.759 *R* = 0.185	K–M analysis: *P* < 0.001 M: *P* = 0.130 Stage: *P* = 0.072 *R* = 0.575	K–M analysis: *P* = 0.021 M: *P* = 0.692 Stage: *P* = 0.930 *R* = 0.469	K–M analysis: *P* < 0.001 M: *P* < 0.001 Stage: *P* < 0.001 *R* = 0.447	K–M analysis: *P* < 0.001 M: *P* = 0.034 Stage: *P* = 0.052 *R* = 0.163
SurvExpress		K–M analysis: *P* = 0.080	K–M analysis: *P* = 0.450	K–M analysis: *P* = 0.083	K–M analysis: *P* = 0.006	K–M analysis: *P* = 0.072	K–M analysis: *P* < 0.001	K–M analysis: *P* = 0.022
Firebrowse		Fold change: 0.885	Fold change: 0.965	Fold change: 0.768	Fold change: 1.31	Fold change: 0.958	Fold change: 2.71	Fold change: 1.47

*calcium binding and coiled-coil domain 1 (CALCOCO1); cold inducible RNA binding protein (CIRBP); prolyl 4-hydroxylase, transmembrane (P4HTM); Retained Intron of Ras Homolog Family Member T2 (RHOT2); TBC1 domain family member 17 (TBC1D17); T-Cell Immune Regulator 1 (TCIRG1); thimet oligopeptidase 1 (THOP1)*.

**Table 2:** The mean H-score of RHOT2 and TCIRG1 in Normal kidney and KIRC.

**Biomarker**	**Normal kidney**	**KIRC**	***p***
RHOT2	2.43	3.48	0.005
TCIRG1	2.03	2.96	0.008

*Retained Intron of Ras Homolog Family Member T2 (RHOT2); T-Cell Immune Regulator 1 (TCIRG1); Kidney renal clear cell carcinoma (KIRC)*.

### Comprehensive Analysis of ASEs and Signaling Pathways

**Figure 8:**
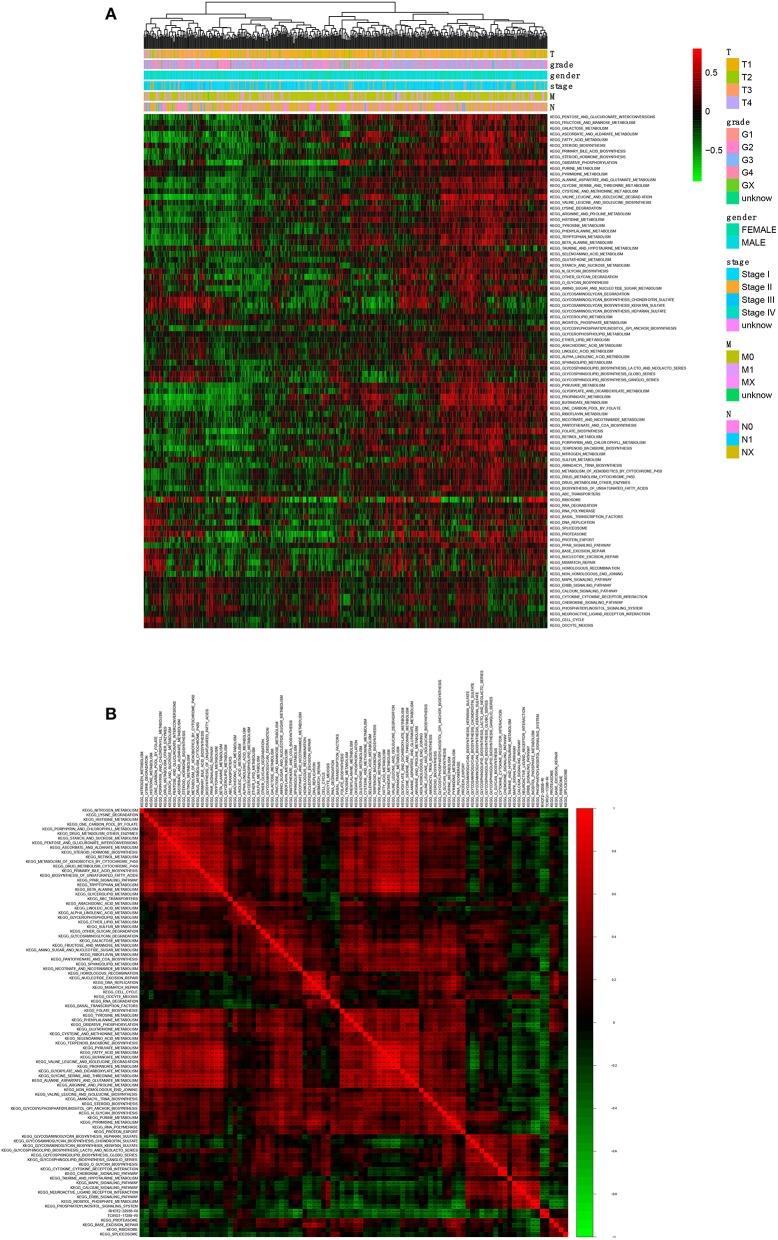
The co-expression analysis between ASEs and signaling pathways. **(A)**: The co-expression heatmap of OS-related KEGG pathways; **(B)** The co-expression heatmap of OS-related KEGG pathways, RHOT2-32938-RI and TCIRG1-17288-RI. KEGG, Kyoto Encyclopedia of Genes and Genomes; RHOT2, Retained Intron of Ras Homolog Family Member T2; TCIRG1, T-Cell Immune Regulator 1.

A total of 90 KEGG pathways were identified as the OS-related KEGG pathways in GSVA and the univariate Cox regression analysis (Figure 8A). As shown, RHOT2–32938–RI was associated with sphingolipid metabolism, N-glycan biosynthesis and glycosphingolipid biosynthesis lacto and neolacto series. TCIRG1-17288-RI was associated with sphingolipid metabolism, purine metabolism and N-glycan biosynthesis (Figure 8B).

## Discussion

KIRC, one of the most prevalent genitourinary malignancies, is often associated with malignant disease progression and poor therapeutic outcomes. Approximately 30 % KIRC patients were found to be metastastic at initial diagnosis (Gupta et al., 2008). Metastatic KIRC evolves from primary KIRC and harbors multiple subpopulations with transcriptomic features, however, the molecular mechanisms of KIRC tumorigenesis and metastasis still remain unclear. In the meantime, effective diagnostic and prognostic biomarkers were still lacking (Song et al., 2018). Recently, aberrant ASs along with corresponding SFs were shown to have great potential value in exploring cancer biology, as both ASEs and SFs have been proved to produce different isoforms of onco-proteins which are associated with cell proliferation, anti-apoptosis and metastasis (Zhang et al., 2019). However, the roles of OS-SEs, SFs and signaling pathways in the tumorigenesis, metastasis, and prognosis of patients with KIRC were not quite clear.

In our study, a total of 6,081 OS-SEs were identified by univariate Cox regression analysis and we constructed a prediction model based on 5 OS-SEs (C4orf19-69001-AT, C16orf13-32924-ES, KIAA0930-62645-AP, FAM120C-89237-AT, UACA-31439-AP) screened by Lasso regression. Compared with previous prediction model of KIRC, the present on had a higher reliability (AUC: 0.788), with less predicters (Song et al., 2019). Additionally, the risk score was proved to be an independent prognostic factor, suggesting the good applicability in clinic for patients with KIRC.

DDX39B, as a DExD RNA helicase, was known to be involved in transportation of mRNA from nuclear to cytoplasm and pre-mRNA splicing (Shen, 2009). Particularly, the ATPase activity of DDX39B played an essential role in unwinding U4/U6 snRNA duplex and connecting U2 snRNP to the pre-mRNA in the process of ASE (Shen et al., 2008). In the present study, DDX39B was the only SF, whose associated OS-SEs were correlated with OS and metastasis by developing the network of OS-SEs and prognosis-related SFs. Similar to our study, mounting evidences regarded DDX39B as an important SF in triggering the progression and metastasis of various cancers. In BioXpress database, the expression level DDX39B was elevated in 75% (12 in 16) types of cancers (Awasthi et al., 2018a,b). Awastthi S et al. found that DDX39B could regulate the transcription and stability of pre-ribosomal RNA, the global translation, cell growth and proliferation. Furthermore, a study regarding to androgen receptor splice variant AR-V7 indicated that DDX39B could serve as the accelerator of AR-V7 mRNA expression and escalated DDX39B could result in resistance to androgen deprivation therapy and poor prognosis in patients with prostate cancer (Nakata et al., 2017).

Among these identified metastasis-associated OS-SEs, the parent genes of RHOT2-32938-RI and TCIRG1-17288-RI were verified by comprehensive databases. RHOT2 gene encodes a member of Rho family of GTPase, which are localized to the outer membrane of mitochondria (Wang et al., 2011). It plays an active role in mitochondrial fusion-fission dynamics, trafficking mitophagy function (Zheng et al., 2015). Mitochondrial dynamics was shown to be reprogrammed in tumor cells via gathering mitochondria at the cortical cytoskeleton (Caino et al., 2016). The mechanism could power the membrane machinery of cell movements, maintained phosphorylation of cell motility kinases, and heightened tumor invasion, chemotaxis, and metastasis (Caino et al., 2016; Agarwal et al., 2019). Besides, remodeling of mitochondrial functions is considered the commonest modified downstream of MYC gene, due to the MYC-dependent transcriptional control of GTPase RHOT1/RHOT2 and posttranslational modifications, such as RHOT phosphorylation by PINK kinase (Wang et al., 2011; Bailey et al., 2018). However, the exact function of RHOT2 has not been explored yet in KIRC. In our study, we found that abnormal expression of ASE of RHOT2 regulated by aberrant DDX39B could result in poor prognosis and tumor metastasis in patients with KIRC. Additionally, we also found out RHOT2-32938-RI was associated with sphingolipid metabolism, N-glycan biosynthesis and glycosphingolipid biosynthesis lacto and neolacto series by co-expression analysis. This might be a novel posttranslational regulation and new function for RHOT2 in KIRC tumorigenesis, progression and metastasis.

TCIRG1 (T-Cell Immune Regulator 1), also known as TIRC7, is essential in T cell activation (Heinemann et al., 1999). Previous studies revealed that TCIRG1 was widely up-regulated in numerous tumors, such as hepatocellular carcinoma, esophageal adenocarcinoma and breast cancer, which might be associated with autophagic sequestration and degradation (Blair and Athanasou, 2004; Hinton et al., 2009; Botelho et al., 2010). With regard to tumor metastasis, TCIRG1 was reported to modulate the EMT regulatory proteins, such as E-cadherin, N-cadherin, Fibronectin, Vimentin, Snail and Slug, and regulate tumor invasion and metastasis in MDA-MB-231, B16-F10, and SNU475 cells (Hinton et al., 2009; Yotsumoto et al., 2013; Yang et al., 2018; Zhou et al., 2018). In our study, we found that aberrant ASE of TCIRG1 was associated with poor prognosis and tumor metastasis in patients with KIRC. In addition, the parent gene TCIRG1 was verified to be associated with OS and metastasis by external database. With regard to KIRC metastasis, bone is one of the most common sites. In this case, patients often experience local pain and even pathological fracture due to osteolytic destruction. As TCIRG1 encodes the osteoclast-specific 116-kD subunit of the vacuolar proton pump and its defect is responsible for a subset of human autosomal recessive osteopetrosis (Frattini et al., 2000), TCIRG1 could be regarded as the potential targets for KIRC metastasis, especially to skeletal metastasis. Nowadays, many anti-TCIRG1 specific monoclonal antibody (mAb) have been developed for different diseases (Kumamoto et al., 2004; Utku et al., 2006). Thus, further cell and animal experiments should be performed to detect the therapeutic effects of anti-TCIRG1 specific mAb in KIRC skeletal metastasis.

To further investigate the deep mechanism of DDX39B regulating RHOT2-32938-RI and TCIRG1-17288-RI, sphingolipid metabolism and N-glycan biosynthesis were identified as the overlapped co-expression signal pathways. Sphingolipids are the major molecules presenting on the cell membranes, which are composed of sphingosine-1-phosphate (S1P) and ceramide (Ogretmen, 2018). Sphingolipids metabolism is pivotal for normal cellular homeostasis with various events, including endocytosis, nutrient transport and protein synthesis. Bioactive sphingolipid could induce cell motility, migration and phenotypic plasticity, which result in tumor invasion and metastasis (Kumamoto et al., 2004; Bonora et al., 2015; Ogretmen, 2018).

N-glycans biosynthesis play important roles in the immune system, pathogen recognition and tumor metastasis via regulating cell adhesion and ligand recognition (Kadam, 2016). The modification of N-glycans also alter cell-cell or cell-matrix contacts and contribute to EMT, invasion and metastasis (Kadam, 2016). E-cadherin, an adhesion molecule, harbors mainly bisecting N-glycans by MGAT3 enzyme in normal epithelial cells. In the tumorigenesis, MGAT3 is down-regulated by promoter methylation and its counterpart MGAT5 is up-regulated. This change results in the formation of tri- and tetraantennary complex glycans on cadherins and E-cadherin internalization to the cytoplasm (Pinho et al., 2013).

There were still some limitations in our study. First of all, this study was a pure bioinformatics study, and the scientific hypothesis was not proved by biological experiments. Second, although the results were verified by external database, the sequencing data were obtained from one single cohort and the sample size was limited. Third, only the primary samples were found in TCGA database and the lack of samples of metastatic sites, such as lung, bone and brain, made this study less complete. At last, the limitation of single omics analysis was also an inherent defect of this study.

In the future, in order to verify the important roles of RHOT2 and TCIRG1 in KIRC metastasis, the functional experiment, such as wound healing and transwell, will be performed in KIRC in which the RHOT2 or TCIRG1 gene has been knocked out (Calabretta et al., 2016; Qi et al., 2016; Chen et al., 2017; Couture et al., 2017; Zhou et al., 2017). Next, these cells will also be used in the nude mouse tumor metastasis model. In addition, a direct mechanism experiment proving the direct mechanism of the splicing factor DDX39B producing splicing isoforms of RHOT2 and TCIRG1 will also be performed. Furthermore, clinical sampes of lung, bone and brain metastasis from KIRC will also be used to detect the expressions of RHOT2 and TCIRG1.

## Conclusions

In conclusion, we established the prediction model with good with good performance in external validation. Based on the comprehensive bioinformatics analysis, we proposed that aberrant DDX39B regulated RHOT2-32938-RI and TCIRG1-17288-RI might be related to the tumorigenesis, metastasis and poor prognosis of KIRC via sphingolipid metabolism or N-glycan biosynthesis pathway.

## Data Availability Statement

All datasets for this study are included in the TCGA-KIRC program.

## Ethics Statement

This study was approved by the Ethics Committee of Tongji Hospital, Tongji University School of Medicine.

## Author Contributions

TM, RH, ZZ, ZH, HY, CJ, PY, PH, XZ, ZL, DS, JZ, and LC: conception, design, manuscript writing, and final approval of manuscript. DS, JZ, and LC: provision of study material. TM, RH, and ZZ: collection and assembly of data. TM, RH, ZZ, ZH, HY, CJ, PY, PH, XZ, and ZL: Data analysis and interpretation.

### Conflict of Interest

The authors declare that the research was conducted in the absence of any commercial or financial relationships that could be construed as a potential conflict of interest.

## Supplementary Material

Supplementary Figure 1 Bubble plots of the top 20 most significant OS-SEs among seven types of splicing patterns: **(A)** AA; **(B)** AD; **(C)** AP; **(D)** AT; **(E)** ES; **(F)** ME; **(G)** RI. OS-SEs, overall survival-related ASEs; AA, alternate acceptor; AD, alternate donor; AP, alternate promoter; AT, alternate terminator; ES, exon skip; ME, mutually exclusive exons; RI, retained intron.

Supplementary Figure 2 The beeswarm plots of CALCOCO1-22108-RI **(A)**, CIRBP-46432-RI **(B)**, P4HTM-64788-ES **(C)**, TBC1D17-51116-ES **(D)**, THOP1-46623-AP **(E)** according to metastasis or not; The beeswarm plots of CALCOCO1-22108-RI **(F)**, CIRBP-46432-RI **(G)**, P4HTM-64788-ES **(H)**, TBC1D17-51116-ES **(I)**, THOP1-46623-AP **(J)** according to clinical status. CALCOCO1, calcium binding and coiled-coil domain 1; CIRBP, cold inducible RNA binding protein; P4HTM, prolyl 4-hydroxylase, transmembrane; TBC1D17, TBC1 domain family member 17; THOP1, thimet oligopeptidase 1.

Supplementary Figure 3 The expressions of RHOT2 and TCIRG1 according to M **(A)** and clinical stage **(B)** in LinkedOmics; The Kaplan-Meier curve of RHOT2 and TCIRG1 in UALCAN **(C)** and SurvExpress **(D)**. RHOT2, Retained Intron of Ras Homolog Family Member T2; TCIRG1, T-Cell Immune Regulator.

## Abbreviations

KIRC: Kidney renal clear cell carcinoma
AS: Alternative splicing
ASEs: alternative splicing events
OS-SEs: overall survival-related ASEs
DDX39B: DExD-Box Helicase 39B
RHOT2: Retained Intron of Ras Homolog Family Member T2
TCIRG1: T-Cell Immune Regulator 1
SFs: splicing factors
EMT: epithelial-mesenchymal transition
GO: Gene Ontology
KEGG: Kyoto Encyclopedia of Genes and Genomes
mAb: monoclonal antibody
S1P: sphingosine-1-phosphate
TCGA: the Cancer Genome Atlas
PSI: percent splicing
GSVA: Gene Set Variation Analysis
IHC: Immunohistochemistry
AUC: Area under curve.
