# Supplementary material for: Identification of Prognostic and Metastatic Alternative Splicing Signatures in Kidney Renal Clear Cell Carcinoma
Source: Front Bioeng Biotechnol. 2019 Oct 15;7:270. doi: 10.3389/fbioe.2019.00270 (PMC6803439; doi:10.3389/fbioe.2019.00270)
Supplement: Supplementary file 1 [file Table_1.DOCX]

**Table 1** The external validation of CALCOCO1, CIRBP, P4HTM, RHOT2, TBC1D17, TCIRG1 and THOP1

calcium binding and coiled-coil domain 1 (CALCOCO1); cold inducible RNA binding protein (CIRBP)；prolyl 4-hydroxylase, transmembrane (P4HTM); Retained Intron of Ras Homolog Family Member T2 (RHOT2); TBC1 domain family member 17 (TBC1D17);T-Cell Immune Regulator 1 (TCIRG1); thimet oligopeptidase 1 (THOP1)

| **Database** | | **CALCOCO1**  **(anti-oncogene)** | **CIRBP**  **(anti-oncogene)** | **P4HTM**  **(anti-oncogene)** | **RHOT2**  **(oncogene)** | **TBC1D17**  **(oncogene)** | **TCIRG1**  **(oncogene)** | **THOP1**  **(oncogene)** |
| --- | --- | --- | --- | --- | --- | --- | --- | --- |
| **UALCAN** | | Expression: p < 0.001  Stage: p < 0.001  K-M analysis: P = 0.010 | Expression: p = 0.023  Stage: p < 0.001  K-M analysis: p = 0.001 | Expression: p < 0.001  Stage: p < 0.001  K-M analysis: p = 0.240 | Expression: p < 0.001  Stage: p < 0.001  K-M analysis: p < 0.001 | Expression: p = 0.010  Stage: p < 0.001  K-M analysis: p = 0.250 | Expression: p < 0.001  Stage: p < 0.001  K-M analysis: p < 0.001 | Expression: p < 0.001  Stage: p < 0.001  K-M analysis: p = 0.410 |
| **The Human Protein Atlas** | | Tumor median  Normal high  K-M analysis P < 0.001 | Tumor low  Normal high  K-M analysis P < 0.001 | Tumor not detected  Normal median  K-M analysis P < 0.001 | Tumor high  Normal median  K-M analysis P < 0.001 | Tumor median  Normal median  K-M analysis P < 0.001 | Tumor high  Normal median  K-M analysis P < 0.001 | Tumor high  Normal high  K-M analysis P < 0.001 |
| **Kaplan Meier**  **plotter** | **Best cutoff** | P < 0.001 | P < 0.001 | P = 0.004 | P < 0.001 | P < 0.001 | P < 0.001 | P = 0.008 |
|  | **Median value** | P = 0.334 | P = 0.121 | P = 0.168 | P = 0.011 | P = 0.216 | P < 0.001 | P = 0.193 |
| **LinkedOmics** | | K-M analysis: P = 0.364  M: P = 0.128  Stage: P = 0.047  R = 0.541 | K-M analysis: P = 0.044  M: P < 0.001  Stage: P < 0.001  R = 0.545 | K-M analysis: P = 0.371  M: P = 0.630  Stage: P = 0.759  R = 0.185 | K-M analysis: P < 0.001  M: P = 0.130  Stage: P = 0.072  R = 0.575 | K-M analysis: P = 0.021  M: P = 0.692  Stage: P = 0.930  R = 0.469 | K-M analysis: P < 0.001  M: P < 0.001  Stage: P < 0.001  R = 0.447 | K-M analysis: P < 0.001  M: P = 0.034  Stage: P = 0.052  R = 0.163 |
| **SurvExpress** | | K-M analysis: P = 0.080 | K-M analysis: P = 0.450 | K-M analysis: P = 0.083 | K-M analysis: P = 0.006 | K-M analysis: P = 0.072 | K-M analysis: P < 0.001 | K-M analysis: P = 0.022 |
| **Firebrowse** | | Fold change: 0.885 | Fold change: 0.965 | Fold change: 0.768 | Fold change: 1.31 | Fold change: 0.958 | Fold change: 2.71 | Fold change: 1.47 |

Table 2. The mean H-score of RHOT2 and TCIRG1 in Normal kidney and KIRC

| Biomarker | Normal kidney | KIRC | p |
| --- | --- | --- | --- |
| RHOT2 | 2.43 | 3.48 | 0.005 |
| TCIRG1 | 2.03 | 2.96 | 0.008 |

Retained Intron of Ras Homolog Family Member T2 (RHOT2); T-Cell Immune Regulator 1 (TCIRG1); Kidney renal clear cell carcinoma (KIRC)
