# Supplementary figures and images for: Identification of Prognostic and Metastatic Alternative Splicing Signatures in Kidney Renal Clear Cell Carcinoma
Source: Front Bioeng Biotechnol. 2019 Oct 15;7:270. doi: 10.3389/fbioe.2019.00270 (PMC6803439; doi:10.3389/fbioe.2019.00270)

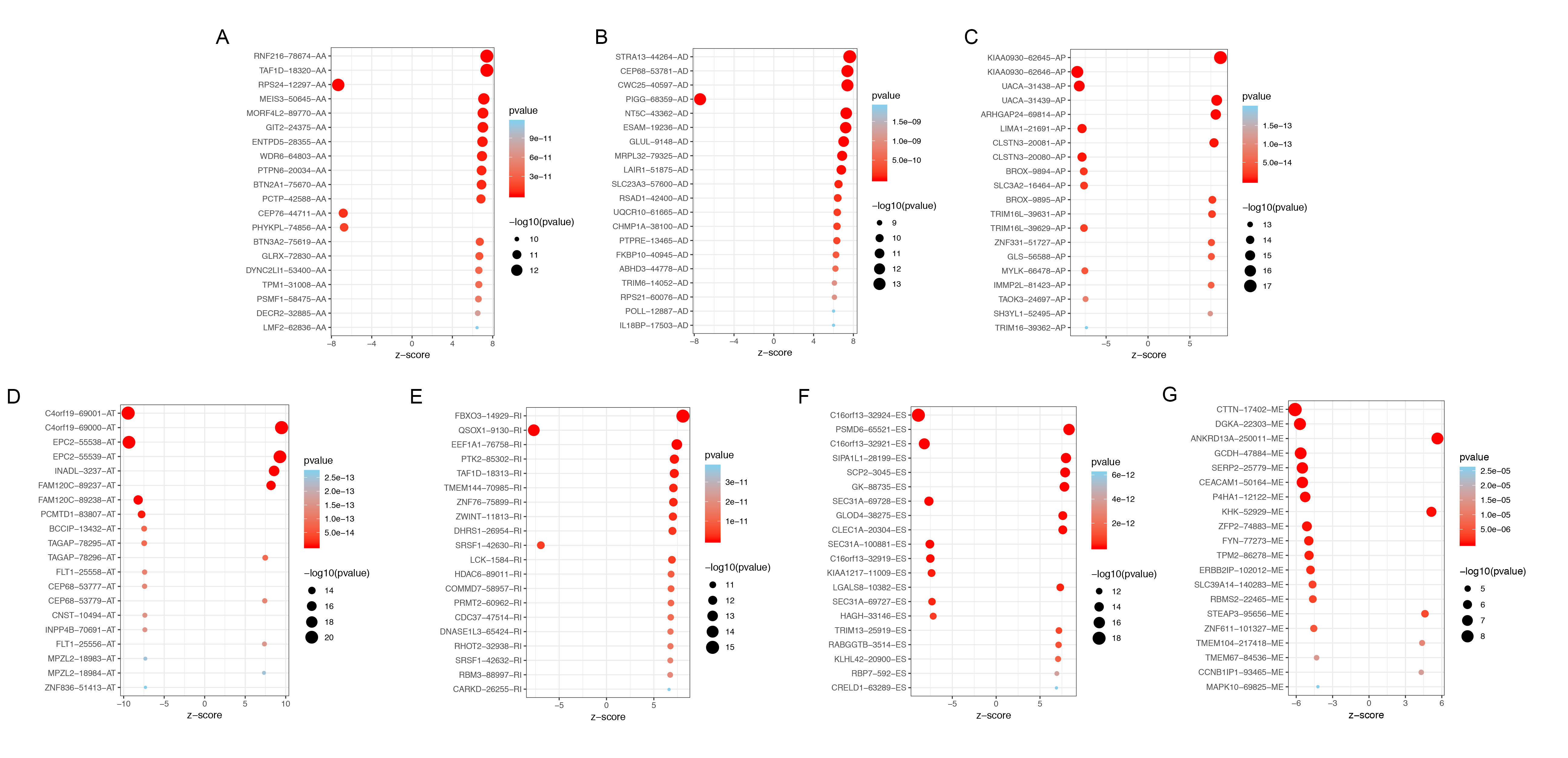

Supplement: Supplementary Figure 1 — Bubble plots of the top 20 most significant OS-SEs among seven types of splicing patterns: (A) AA; (B) AD; (C) AP; (D) AT; (E) ES; (F) ME; (G) RI. OS-SEs, overall survival-related ASEs; AA, alternate acceptor; AD, alternate donor; AP, alternate promoter; AT, alternate terminator; ES, exon skip; ME, mutually exclusive exons; RI, retained intron. [file Image_1.TIF]

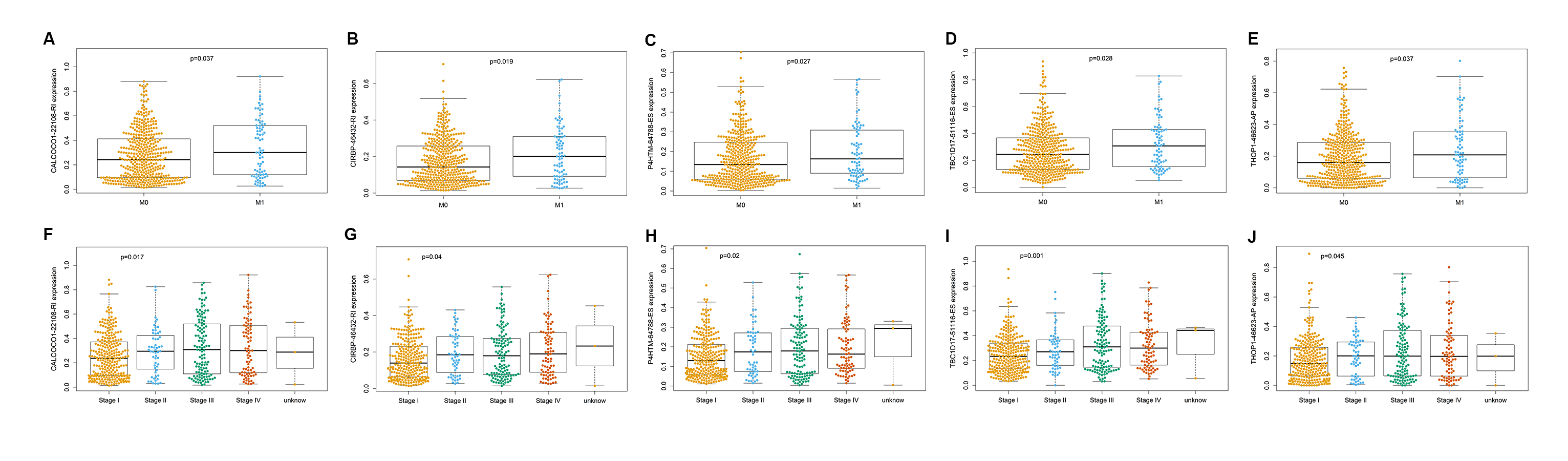

Supplement: Supplementary Figure 2 — The beeswarm plots of CALCOCO1-22108-RI (A), CIRBP-46432-RI (B), P4HTM-64788-ES (C), TBC1D17-51116-ES (D), THOP1-46623-AP (E) according to metastasis or not; The beeswarm plots of CALCOCO1-22108-RI (F), CIRBP-46432-RI (G), P4HTM-64788-ES (H), TBC1D17-51116-ES (I), THOP1-46623-AP (J) according to clinical status. CALCOCO1, calcium binding and coiled-coil domain 1; CIRBP, cold inducible RNA binding protein; P4HTM, prolyl 4-hydroxylase, transmembrane; TBC1D17, TBC1 domain family member 17; THOP1, thimet oligopeptidase 1. [file Image_2.TIF]

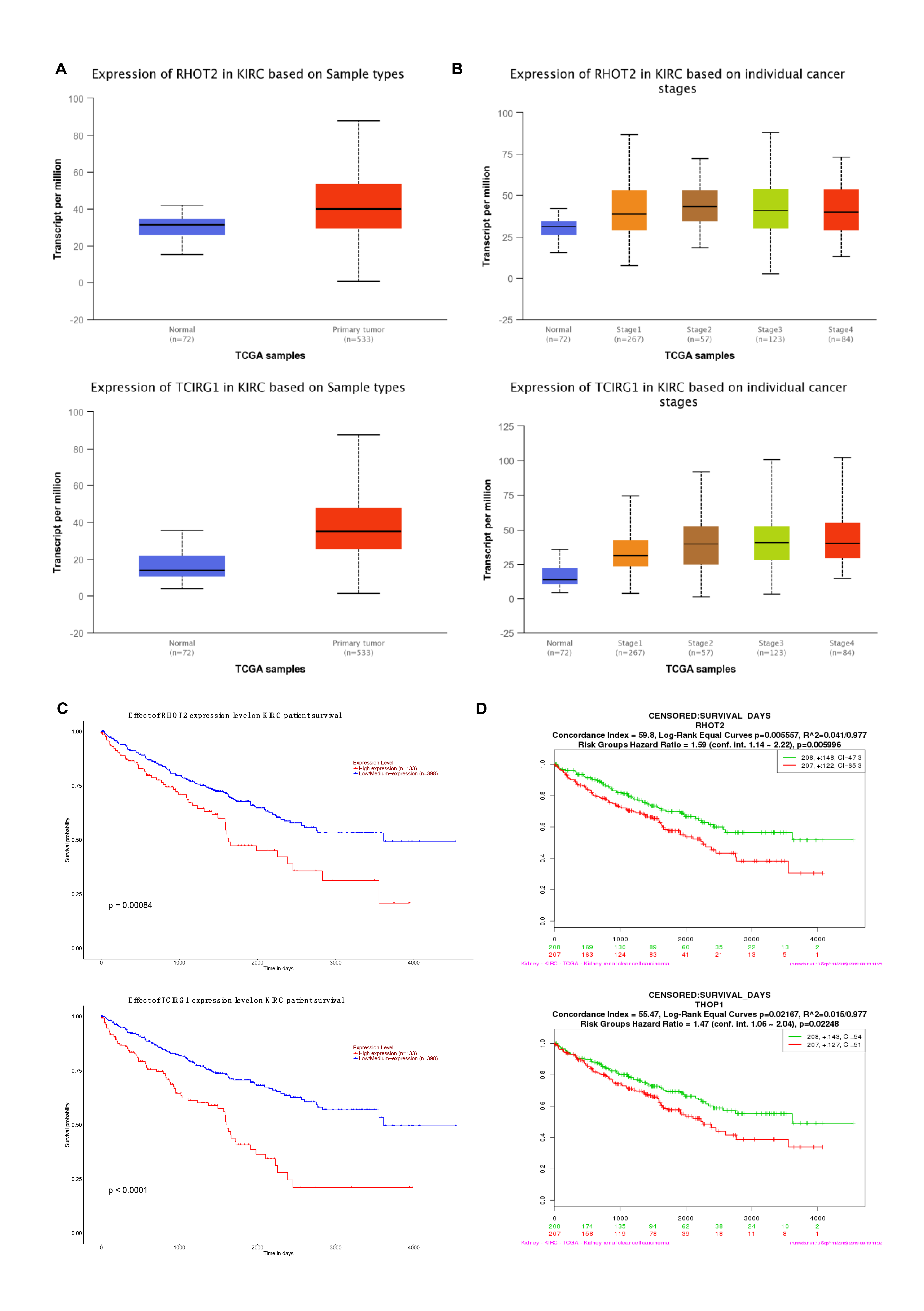

Supplement: Supplementary Figure 3 — The expressions of RHOT2 and TCIRG1 according to M (A) and clinical stage (B) in LinkedOmics; The Kaplan-Meier curve of RHOT2 and TCIRG1 in UALCAN (C) and SurvExpress (D). RHOT2, Retained Intron of Ras Homolog Family Member T2; TCIRG1, T-Cell Immune Regulator. [file Image_3.TIF]
